# An Italian prospective multicenter survey on patients suspected of having non-celiac gluten sensitivity

**DOI:** 10.1186/1741-7015-12-85

**Published:** 2014-05-23

**Authors:** Umberto Volta, Maria Teresa Bardella, Antonino Calabrò, Riccardo Troncone, Gino Roberto Corazza

**Affiliations:** 1 Department of Medical and Surgical Sciences, University of Bologna, Bologna, Italy; 2 IRCCS Ca’ Granda, University of Milan, Milano, Italy; 3 Gastroenterology Unit, University of Florence, Firenze, Italy; 4 Department of Pediatrics, University Federico II, Naples, Italy; 5 First Department of Internal Medicine, St. Matteo Hospital, University of Pavia, Pavia, Italy

**Keywords:** Non-celiac gluten sensitivity, Celiac disease, Prospective survey, Clinical picture, Duodenal biopsy, Anti-gliadin antibodies

## Abstract

**Background:**

Non-celiac gluten sensitivity (NCGS) is still an undefined syndrome with several unsettled issues despite the increasing awareness of its existence. We carried out a prospective survey on NCGS in Italian centers for the diagnosis of gluten-related disorders, with the aim of defining the clinical picture of this new syndrome and to establish roughly its prevalence compared with celiac disease.

**Methods:**

From November 2012 to October 2013, 38 Italian centers (27 adult gastroenterology, 5 internal medicine, 4 pediatrics, and 2 allergy) participated in this prospective survey. A questionnaire was used in order to allow uniform and accurate collection of clinical, biochemical, and instrumental data.

**Results:**

In total, 486 patients with suspected NCGS were identified in this 1-year period. The female/male ratio was 5.4 to 1, and the mean age was 38 years (range 3–81). The clinical picture was characterized by combined gastrointestinal (abdominal pain, bloating, diarrhea and/or constipation, nausea, epigastric pain, gastroesophageal reflux, aphthous stomatitis) and systemic manifestations (tiredness, headache, fibromyalgia-like joint/muscle pain, leg or arm numbness, 'foggy mind,' dermatitis or skin rash, depression, anxiety, and anemia). In the large majority of patients, the time lapse between gluten ingestion and the appearance of symptoms varied from a few hours to 1 day. The most frequent associated disorders were irritable bowel syndrome (47%), food intolerance (35%) and IgE-mediated allergy (22%). An associated autoimmune disease was detected in 14% of cases. Regarding family history, 18% of our patients had a relative with celiac disease, but no correlation was found between NCGS and positivity for HLA-DQ2/-DQ8. IgG anti-gliadin antibodies were detected in 25% of the patients tested. Only a proportion of patients underwent duodenal biopsy; for those that did, the biopsies showed normal intestinal mucosa (69%) or mild increase in intraepithelial lymphocytes (31%). The ratio between suspected NCGS and new CD diagnoses, assessed in 28 of the participating centers, was 1.15 to 1.

**Conclusions:**

This prospective survey shows that NCGS has a strong correlation with female gender and adult age. Based on our results, the prevalence of NCGS seems to be only slightly higher than that of celiac disease.

Please see related article http://www.biomedcentral.com/1741-7015/12/86.

## Background

In recent years, a growing number of subjects worldwide have reported that ingestion of wheat and, to a lesser extent, of other cereals such as barley, rye, and spelt cause them intestinal and extraintestinal symptoms without the diagnostic features of celiac disease (CD) or wheat allergy (WA) [1]. This self-reported wheat sensitivity has fueled the debate on the possible existence of a new syndrome, which has been recently named as non-celiac gluten sensitivity (NCGS) to differentiate it from CD [2-4]. Gluten, the main protein complex contained not only in wheat, but also in barley, rye, and spelt, has been identified as the possible trigger of this syndrome. The definition of NCGS has been questioned as possibly being too restrictive [5]. Indeed, it has been suggested that wheat proteins other than gluten, such as amylase trypsin inhibitors, can contribute to this disorder [6]. In addition, recent studies have emphasized the possible role of fermentable oligosaccharides, monosaccharides and disaccharides and polyols (FODMAPs) in the development of this syndrome [7]. Interestingly, together with milk, legumes, honey, some fruits (watermelon, cherry, mango, pear) and some vegetables (chicory, fennel, beetroot, and leek), the most common food sources of FODMAPs also include wheat and rye, the same grains and cereals that also contain gluten proteins [8]. Despite awareness of these limitations, for the time being the acronym NCGS has been accepted by the scientific community, and it is commonly used to indicate this syndrome, as reported in the majority of published papers [2,3,9].

The prevalence of NCGS is far from being defined, with only few available data, which are largely variable. In the National Health and Nutrition Examination Survey, a primary care program designed to assess the health and nutritional status of people in the USA, 49 cases of suspected NCGS (0.6%) were identified in 7,762 subjects in the period 2009 to 2010 [10], whereas at the University of Maryland, a tertiary care center for the diagnosis of gluten-related disorders, the criteria for NCGS were recognized in 347 (6%) of 5,896 patients observed between 2004 and 2010 [11].

The Italian Celiac Disease Association (AIC) together with the Italian Coeliac Foundation (FC) promoted a prospective multicenter survey on NCGS. The purpose of this study was to provide a picture of patients with suspected NCGS seen in Italian centers skilled in the diagnosis of gluten-related disorders. An additional aim was to define the ratio between suspected NCGS cases and new CD diagnoses in order to get a rough idea of their frequencies.

## Methods

This prospective survey study was approved by the Ethical Committee of S.Orsola-Malpighi Hospital in November 2012 (Authorization N° 243/2012/O/OssN). An informed consent was obtained from each patient participating in the study.

In total, 38 Italian centers (all recognized as referral centers and included in the register of the Italian Health Ministry for the diagnosis of gluten-related disorders) participated in this study. Of these 38 centers, 27 were centers of adult gastroenterology, 5 of internal medicine, 4 of pediatrics, and 2 of allergy. The survey started in November 2012 and ended in October 2013, with a duration of 12 months. The design of the prospective survey was planned by the members (UV, MTB, AC, RT, GRC) of the AIC/FC scientific Board for NCGS, coordinated by the authors UV (University of Bologna, Italy) and GRC (University of Pavia, Italy). The study was carried out by all the 38 investigators in charge of their own referral centers for the diagnosis of gluten-related disorders. All patients seen in the outpatient clinics were evaluated by skilled investigators, who identified subjects with suspected NCGS. The diagnosis of NCGS was made when the patient reported intestinal and extraintestinal symptoms occurring after the ingestion of gluten, which improved or disappeared when gluten was withdrawn from the diet, and recurred when gluten was reintroduced into the diet. Because there are as yet no biomarkers available, NCGS can be suspected only on clinical grounds, after the exclusion of CD and WA, based on negativity for anti-tissue transglutaminase (anti-tTG) antibodies and anti-endomysial antibodies (EmA) of the IgA class (for CD), and negativity for specific IgE antibodies to wheat and/or skin prick tests (for WA). The diagnostic criteria for NCGS have been extensively quoted in the literature [11,12]. In order to facilitate accurate collection of clinical, biochemical and instrumental data, an NCGS questionnaire (Additional file 1) was devised by the AIC/FC Board for NCGS. The questionnaire included 60 items/questions to obtain a set of information, including age and gender, gastrointestinal and extraintestinal symptoms, symptom frequency, and time interval between the ingestion of gluten and symptom occurrence, who was the first to suspect NCGS (for example, the patient, doctor, friend), associated disorders, positivity for serum specific IgE or prick tests to food and inhalants, family history of CD, serology for anti-gliadin antibodies of first (AGA) and second generation (deamidated gliadin peptide; DGP) of the IgG and IgA classes, biochemical abnormalities, human leukocyte antigen (HLA) typing, and intestinal biopsy (when performed). The questionnaire was filled in for each suspected NCGS case by the main investigator of each participating center. Once completed, the questionnaire was entered in an online platform; data were processed by BM (AIC Observatory) and evaluated by the two coordinators (UV and GRC).

In addition, 28 out of the 38 participating centers also provided, together with the total number of suspected NCGS cases, the total number of new CD diagnoses and of patients seen in the period of the study, in order to establish the ratio of NCGS diagnoses to total number of patients seen, CD diagnoses to number of total patients seen and NCGS to CD. In total, 12,255 consecutively observed patients were clinically evaluated by the investigators, and underwent thorough diagnostic investigation, including blood tests and invasive procedures (when needed) in order to confirm or exclude NCGS and CD.

## Results

**Figure 1:**
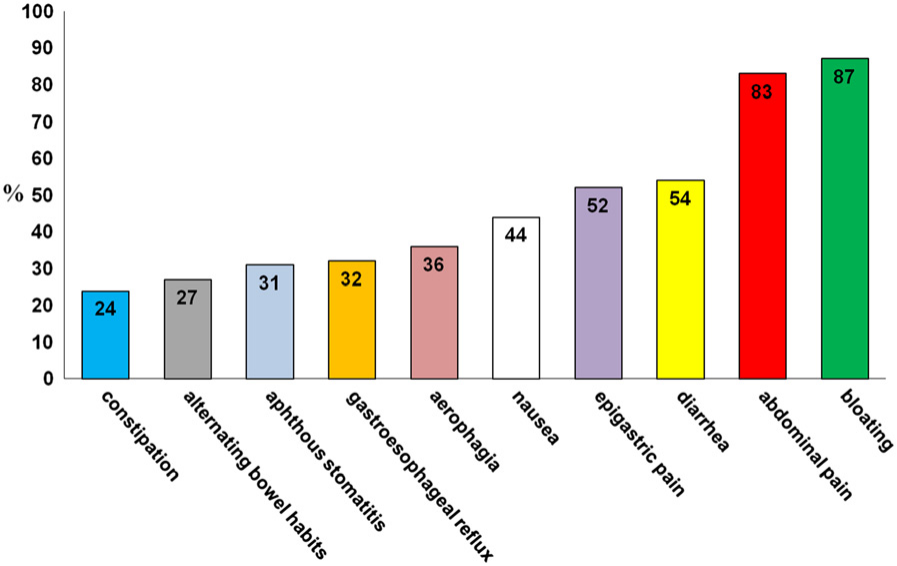
**Gastrointestinal symptoms in suspected non-celiac gluten sensitivity (NCGS).** Prevalence (expressed as percentage) of gastrointestinal manifestations in the 486 patients with suspected NCGS identified in 38 Italian centers skilled in the diagnosis of gluten-related disorders.

**Figure 2:**
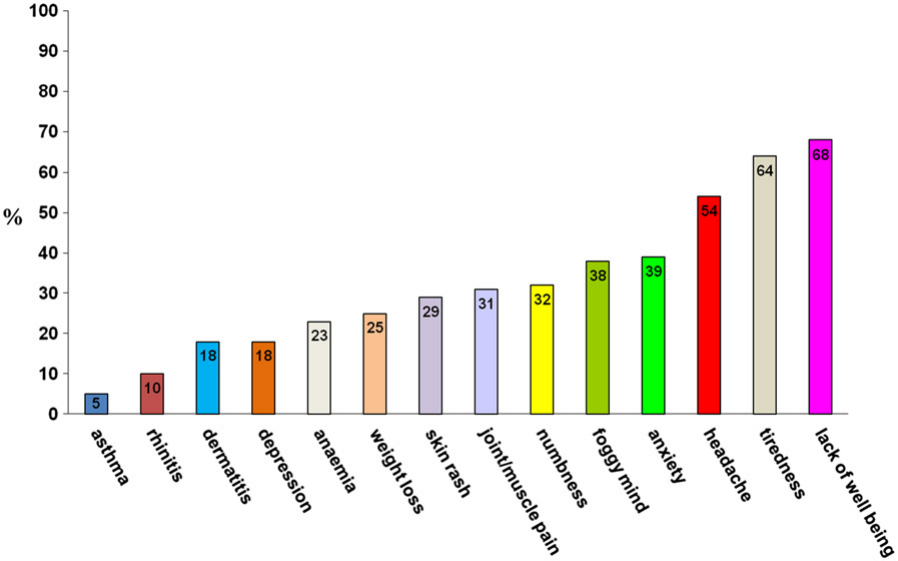
**Extraintestinal manifestations in suspected non-celiac gluten sensitivity (NCGS).** Prevalence (expressed as percentage) of extraintestinal manifestations in the 486 patients with suspected NCGS identified in 38 Italian centers skilled in the diagnosis of gluten-related disorders.

During the 12 months of the prospective survey, the 38 participating centers (of which only 4 were pediatric centers) identified 486 patients with suspected NCGS. Of these, 410 were female (84%) with a female to male ratio of 5.4 to 1. The mean age was 38 years (range 3 to 81 years). The large majority of patients reported more than two associated gastrointestinal or extraintestinal symptoms (Figure 1, Figure 2). Of the gastrointestinal symptoms, the most frequent were bloating and abdominal pain, found in 87% and in 83%, respectively, of the patients with suspected NCGS. More than 50% of patients reported diarrhea, with the number of evacuations per day ranging from 3 to 10, while 27% had alternating bowel habits and 24% had constipation. After bloating and abdominal pain, epigastric pain was the most frequent symptom, being found in 52% of patients, followed with decreasing prevalence by nausea, aerophagia, gastroesophageal reflux disease, and aphthous stomatitis. The most frequent extraintestinal manifestations were tiredness and lack of well being, reported by 64% and 68%, respectively, of the enrolled subjects. In addition, a high prevalence of neuropsychiatric symptoms including headache (54%), anxiety (39%), 'foggy mind' (38%), and arm/leg numbness (32%) were recorded. Other extraintestinal manifestations emerging from the analysis of the survey responses were joint/muscle pain resembling fibromyalgia (31%), weight loss (25%), anemia (due both to iron deficiency and low folic acid; 22%), depression (18%), dermatitis (18%) and skin rash (29%,). Less than 10% of patients showed a clinical picture of allergic manifestations such as asthma or rhinitis.

**Figure 3:**
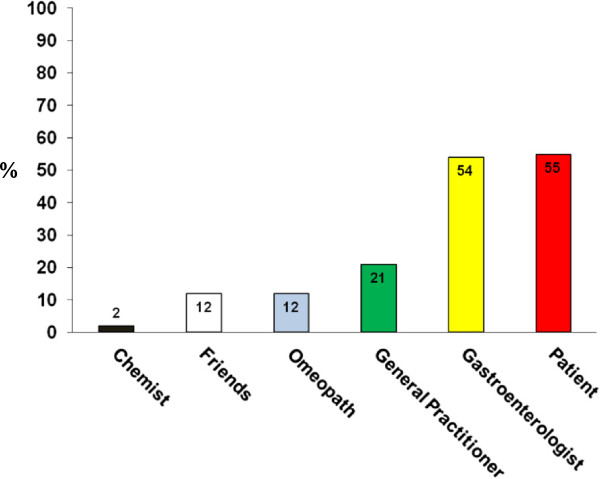
**Who was the first to suspect non-celiac gluten sensitivity (NCGS)?** Prevalence (expressed as percentage) of the person who firstly suspected the existence of NCGS in the 486 patients hypothesized to suffer from this syndrome.

**Figure 4:**
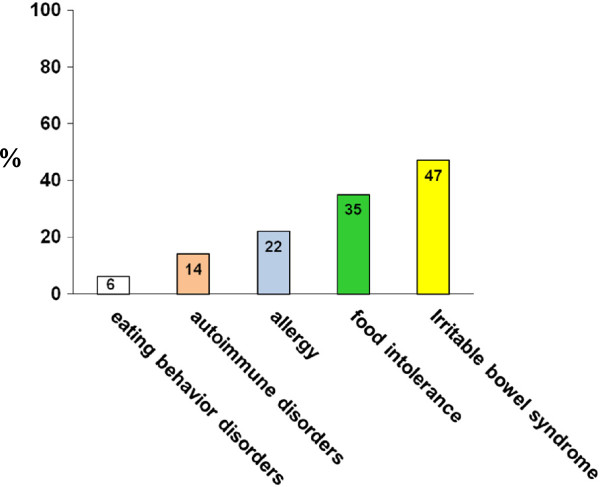
**Disorders associated to suspected non-celiac gluten sensitivity (NCGS).** Prevalence (expressed as percentage ) of associated disorders detected in the 486 patients with suspected NCGS.

In the study, 95% of patients with suspected NCGS reported the appearance of the aforementioned symptoms every time or often after the ingestion of gluten-containing food. In more than half of these patients, the symptoms occurred within 6 hours after gluten ingestion; in about 40%, between 6 and 24 hours after ingestion; and in less than 10%, more than 24 hours after ingestion. Symptoms appeared several months before the diagnosis of NCGS was suspected in about 90% of cases, whereas the existence of NCGS was hypothesized within 1 month from its clinical onset in less than 2% of cases.The existence of NCGS was suspected by the patients themselves or by gastroenterologists in more than 50% of our cases, followed by the patient's general practitioner (21%), homeopath/alternative medicine practitioner (12%), friends (12%), or chemist (2%) (Figure 3).The most frequent associated disorder in patients with suspected NCGS was irritable bowel syndrome (IBS) (diagnosed according to Rome III criteria), detected in 47% of cases. About 35% of patients had a previous diagnosis of food intolerance, represented in most cases by lactose intolerance. More than 20% of suspected patients with NCGS patients had an allergy to one or more inhalants, food, or metals, the most frequent being mites (26%), graminaceae (24%), nickel (15%), cat/dog hair (13%), shellfish (8%), and parietaria pollen (6%). About a half of patients with positive allergic tests had high levels of total serum IgE. Eight graminaceae-positive patients showed a secondary IgE response to wheat, always at a very low titer and without clinical manifestations of WA. One or more associated autoimmune diseases were present in 14% of patients, mainly represented by autoimmune thyroiditis, detected in two-thirds of the NCGS autoimmune subgroup. Psoriasis and Graves’ disease were the other two autoimmune disorders observed more frequently, whereas myasthenia gravis, atrophic autoimmune gastritis, scleroderma, type 1 diabetes mellitus, Crohn’s disease and IgA deficiency were sporadically present in these patients (Figure 4). A clinical history of eating behavior and psychiatric disorders preceded the suspected diagnosis of NCGS in 6% of cases.

**Figure 5:**
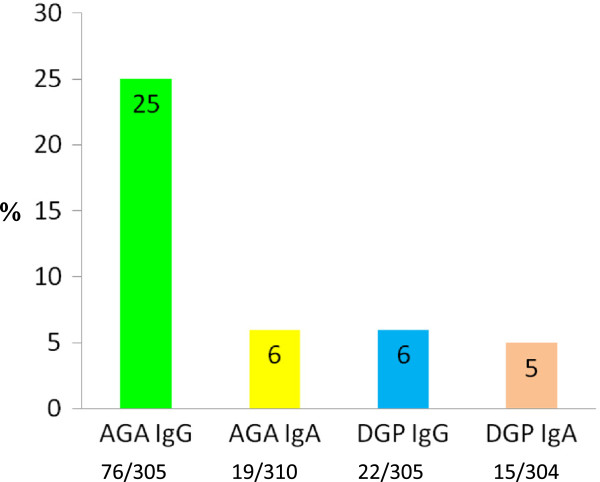
**Immune response to gliadin in suspected non-celiac gluten sensitivity (NCGS).** Prevalence of anti-gliadin antibodies of first (AGA) and second generation (deamidated gliadin peptide; DGP) of the IgG and IgA classes in the 486 patients with suspected NCGS.

Regarding family history, 18% of patients with suspected NCGS had a first or second degree relative affected by CD. No correlation was found with HLA-DQ2 and DQ8, found in 25% and 8% of patients, respectively.As for the immune response to gliadin, AGA IgG were the most frequent serological marker, being found in 76 (25%) of the 305 patients tested, whereas AGA IgA, DGP IgG, and DGP IgA were positive in 6%, 6%, and 5% of the cases studied, respectively (Figure 5).

Laboratory tests displayed low levels of ferritin, folic acid, and vitamin D in 23%, 5%, and 11%, respectively, of patients with suspected NCGS.

Duodenal biopsy was performed in 302 (62%) of the 486 patients, with the finding of Marsh 0 in 209 cases (69%) and Marsh 1 in 93 cases (31%) according to the modified Marsh-Oberhüber classification [13].

As specified in the Materials and Methods section, 28 centers (of which 4 were pediatric centers) reported the total number of suspected NCGS cases, new CD diagnoses, and patients observed during the period of the study. Suspected NCGS and new CD diagnoses were reported for 391 (3.19%) and 340 (2.77%), respectively of the total of 12,255 patients, with a ratio of NCGS to CD of 1.15 to 1. In the four pediatric centers, the number of suspected NCGS cases compared with new CD cases was far lower, with a ratio of NCGS to CD of 0.29 to 1. When we excluded the pediatric centers, the ratio of NCGS to CD was 1.25 to 1 in the remaining 24 centers (Table 1).

**Table 1:** Ratio between suspected NCGS, new CD and patients seen in 28 of the 38 Italian centers participating in the study

	**All 28 centers**	**4 pediatric centers**	**The other 24 centers**^**a**^
NCGS/patients observed	391/12255	11/410	380/11845
	3,19%	2,68%	3.21%
CD/patients observed	340/12255	38/410	302/11845
	2,77%	9,26%	2,55%
NCGS/CD	391/340	11/38	380/302
	1,15:1	0,29:1	1,25:1

CD, celiac disease; NCGS, non-celiac gluten sensitivity.

^a^Internal Medicine, Gastroenterology, Allergy.

## Discussion

NCGS is still an undefined syndrome, with several unsettled issues despite the increasing awareness of its existence [14]. One of the most critical aspects is the absence of biomarkers, making it difficult to reach a clear-cut diagnosis. Consequently, NCGS can be suspected only on the basis of clinical response to gluten ingestion and withdrawal, followed by gluten challenge after both CD and WA have been excluded. The gold-standard assay for confirming NCGS requires dietary elimination, followed by a double-blind, randomized, placebo-controlled (DBPC) food challenge. This procedure is difficult to adopt routinely in clinical practice, and to date, only a few DBPC studies have been performed, showing that in a variable proportion of patients classified as having NCGS, their symptoms were caused by a nocebo effect [15-18]. Indeed, in a number of patients classified as being gluten-sensitive and put on a gluten-free diet (GFD), the double-blind reintroduction of gluten did not result in recurrence of the symptoms, which were instead evoked by the ingestion of placebo. The results of DBPC studies have been controversial and, in some cases, even contradictory, with the same authors who in a first study validated the existence of this syndrome with the central role of gluten as trigger concluding after more recent research that this syndrome is unrelated to gluten and was improved by a low-FODMAPs diet [16,18].

The above data emphasize how difficult it is to be sure of NCGS diagnosis and, consequently, how its prevalence in the general population can only be defined empirically. Indeed, studies in USA have reported a large variability for NCGS prevalence with figures ranging from 0.6% in primary care to 6% in tertiary care [10,11]. This high figure of NCGS is likely to be influenced by the fact that many patients decide to avoid wheat and gluten themselves without taking medical advice, because they feel better if they do not eat gluten-containing foods. In one recent study, a reassessment of patients who had decided by themselves to avoid gluten allowed an alternative diagnosis to be made in about 30% of cases; these diagnoses included small intestinal bacterial overgrowth, fructose or lactose intolerance, microscopic colitis, gastroparesis, and pelvic floor dysfunction [19]. In a recent paper from the UK, it was stated that the self-reported prevalence for NCGS was 13% [20]. The mean age of individuals with self-reported gluten sensitivity was 39.5 years (range 18 to 75 years), and 79% were females. About 5% of children and adolescents in New Zealand were reported to be avoiding gluten in their diet, without any evidence for NCGS in many of these cases [21]. Therefore, it is likely that the number of people worldwide following a GFD is far higher than that of patients with true NCGS.

Previous papers on NCGS have shown that this syndrome is much more frequent in adulthood than in childhood, and also in female than in male patients, with a clinical picture characterized by a large combination of both gastrointestinal and extraintestinal symptoms [2,12,17,22].

To our knowledge, our study is the first multicenter prospective survey on patients with suspected NCGS, and it provides a picture of this syndrome in Italian centers for gluten-related disorder diagnosis. On one hand, it must be emphasized that this study has major limitations because the diagnosis of NCGS was highly presumptive in all our patients, being based exclusively on clinical criteria and on exclusion of CD and WA. None of our patients classified as having NCGS has had his/her diagnosis confirmed by the gold-standard method of testing (DBPC) and, as already reported, it is likely that a proportion of patients identified as suspected NCGS improved after GFD only because of the placebo effect. However, the strengths of this study include the large number of patients investigated and the high skill level of the physicians working in the referral centers involved in this survey. All the patients were prospectively evaluated clinically by experts who planned the diagnostic investigation based on blood tests and endoscopy to identify CD or NCGS.

Our study has a number of important findings. First, our results confirm that suspected NCGS is much more frequent in females than in males (5.4 to 1, respectively), and that this syndrome can have its onset at any age, but is much more common in adulthood, with the maximum peak in the fourth decade of life. Based on our data, suspected NCGS was found in 3.19% of a clinical group at risk for gluten-related disorders. Obviously, this prevalence does not reflect that of suspected NCGS in the general population, but, because the established prevalence of CD in the general population is 1%, and the ratio between suspected NCGS and CD in our study was 1.15 to 1, we can extrapolate that the likely NCGS prevalence in the general population should be slightly higher than 1%. In the four participating pediatric centers, the prevalence of suspected NCGS was far lower than that of CD (0.29 to 1), confirming that this syndrome is rare in childhood [22]. The clinical picture of NCGS emerging from our study is a combination of IBS-like symptoms, including abdominal pain, bloating, bowel habit abnormalities (either diarrhea or constipation), and systemic manifestations such as tiredness, headache, fibromyalgia-like joint/muscle pain, leg or arm numbness, 'foggy mind', dermatitis or skin rash, depression, anxiety, aphthous stomatitis, and anemia (both iron and folate deficiency anemia). From a clinical point of view it is relatively difficult to differentiate NCGS from CD because both conditions share the same clinical features. In the large majority of our patients with suspected NCGS, the time lapse between gluten ingestion and the occurrence of symptoms varied from a few hours to 1 day, whereas in CD this interval is much longer (up to weeks or years). In line with the increased awareness of NCGS by specialists, it should be emphasized that the diagnosis was suspected by a gastroenterologist in a large number of cases. However, in more than half of the cases, NCGS was a self-diagnosis made by the patients, who started to avoid gluten in their diet by themselves. In such cases, gluten reintroduction for at least 6 weeks is highly recommended in order to detect clear-cut histological changes consistent with CD. Recently, it has been suggested that a 3-day gluten challenge can disclose possible CD in patients with self-prescribed GFD by evaluating HLA-DQ2-tetramer staining for gluten-specific T cells [23]. Because HLA typing displays an almost absolute negative predictive value for CD, the absence of HLA-DQ2 and DQ8 can exclude CD in patients with suspected NCGS without the need of gluten challenge [12].

As already emphasized by many papers [16,17,24], NCGS is closely related to IBS, which was found in about half of our NCGS subjects. In addition to IBS, one-third of patients with suspected NCGS displays another food intolerance (most frequently lactose intolerance). A subgroup of patients with suspected NCGS (around 20%) have an IgE-mediated allergy with specific IgE to food and inhalants, the most frequent allergens involved being mites, graminaceae, cat/dog hair, and shellfish. Similar to patients with CD, a relatively high number of patients with suspected NCGS develops nickel allergy. Published studies have previously reported that patients with suspected NCGS did not seem to present major autoimmune comorbidities [11,12]. Unlike these reports, our present study highlights that there is a high prevalence of Hashimoto thyroiditis in patients with suspected NCGS and that other autoimmune conditions such as psoriasis and Graves’ disease seem to be relatively common in these patients. In this respect, NCGS might behave in a similar way to CD, which is characterized by a close association with a wide spectrum of autoimmune disorders.

As already demonstrated [12], NCGS was commonly detected in first-degree relatives of celiacs. However, the genetic typing excluded any relationship between NCGS and HLA-DQ2 and/or HLA-DQ8, which were found in patients with suspected NCGS at a prevalence very close to that of the general population. Interestingly, laboratory findings indicated that more than 20% of patients with suspected NCGS showed biochemical signs of malabsorption, with low levels of ferritin or folic acid or vitamin D. These biochemical deficiencies, which were less pronounced than in CD, might be due to small bowel inflammation reported in NCGS [2,14]. In line with this hypothesis, an increased number of intraepithelial lymphocytes, consistent with a type 1 lesion according to Marsh-Oberhuber classification, has been documented in the intestinal biopsy of about 30% of patients with suspected NCGS. In the majority of the suspected NCGS subgroup identified at Bologna center (by UV), a typical linear distribution of CD3+ T lymphocytes in the deeper part of mucosa and clusters of CD3+ T lymphocytes in the superficial epithelium were observed, in accordance with the pattern identified as a possible histological marker of NCGS [25].

Although no biomarker has been identified to date, previous studies have reported that about 50% of patients with suspected NCGS show positivity for AGA, mainly of the IgG class [11,12,17]. These antibodies are not specific for NCGS, being also found in CD, autoimmune liver disorders, connective tissue disease and IBS,as well as in healthy controls [26], but their finding in patients with a clinical picture consistent with NCGS has been regarded as an element supporting this diagnosis [27]. In this study, AGA IgG were confirmed as the most frequent serological marker found in patients with suspected NCGS, but AGA prevalence was lower than that reported in previous papers (25% vs 50%) [11,12,17]. The lower AGA finding in our study can be explained by the fact that many of our patients had diagnosed themselves with a gluten disorder, thus they were already on a GFD when tested, and AGA tend to disappear very quickly in patients with NCGS after GFD [28]. Positivity for AGA IgA, DGP IgG and DGP IgA were found in only a few patients (5% to 6%).

## Conclusions

The present study provides a picture of suspected NCGS, but we must emphasize the major limitation of the study, namely that the diagnosis of NCGS remained highly presumptive in all our patients, because it was not established by a confirmatory DBPC. A proportion of patients classified as suspected NCGS might improve after GFD only because of the placebo effect. However, even with its limitations, this prospective survey indicates that NCGS may be a frequent finding among patients referred to centers for the diagnosis of gluten-related disorders. This study confirms that this new gluten-induced syndrome appears to have a strong correlation with female gender and adult age, and has a prevalence that might be slightly higher than that of CD, but is certainly far lower than that hypothesized on the basis of previous reports.

## Abbreviations

AGA: Anti-gliadin antibodies; Anti-tTG: Anti-tissue transglutaminase antibodies; CD: Celiac disease; DGP: Deamidated gliadin peptide antibodies; EmA: Anti-endomysial antibodies; FODMAPs: Fermentable oligo-, di-, mono-saccharides and polyols; GFD: Gluten-free diet; HLA: human leukocyte antigen; IBS: Irritable bowel syndrome; NCGS: Non-celiac gluten sensitivity; WA: Wheat allergy.

## Competing interests

The authors declare that they have no competing interests.

## Authors’ contributions

UV: Study design, clinical evaluation, and enrollment of patients, data analysis, writing of the manuscript, and final supervision. MTB: Study design, clinical evaluation and enrollment of patients, and final supervision of the manuscript. AC and RT: Study design and final supervision of the manuscript. GRC: Study design, clinical evaluation and enrollment of patients, data analysis, and final supervision of the manuscript. All authors read and approved the final manuscript. The Study Group for Non-Celiac Gluten Sensitivity, included Carmela Bagnato, Claudio Belcari, Antonella Bellantoni, Giacomo Caio, Francesca Calella, Maria Cappello, Carolina Ciacci, Cinzia D’Agate, Italo De Vitis, Antonio Di Sabatino, Gianmarco Fava, Maria Rita Frau, Alessandro Fugazza, Stefano Andrea Grassi, Giuseppina Larcinese, Giovanni Latella, Adriano Lauri, Angelo Lauria, Nicoletta Lenoci, Norma Beatriz Lopez Rios, Donatella Macchia, Mauro Minelli, Beba Molinari, Olivia Morelli, Maria Gloria Mumolo, Giovanni Niccoli, Caterina Pacenza, Francesco Pallone, Fabrizio Parente, Raffaella Pulitanò, Rossella Pumpo, Gabriele Riegler, Antonio Rispo, Flavio Romolo Rispoli, Francesco Tedone, Flavio Valiante, Giovanni Viviani, and Paolo Usai Satta. All these authors performed clinical evaluation and enrollment of patients, as well as final supervision of the manuscript.

## Authors’ information

Study Group for Non-Celiac Gluten Sensitivity: Carmela Bagnato (Gastroenterology, Madonna delle Grazie, Hospital, Matera), Claudio Belcari (Internal Medicine, Civil Hospital, Pontedera), Antonella Bellantoni (Pediatrics, Bianchi-Melacrino.Morelli Hospital, Reggio Calabria), Giacomo Caio (Dept. Medical and Surgical Sciences, University of Bologna), Francesca Calella (Gastroenterology, S. Giuseppe Hospital, Empoli), Maria Cappello (Gastroenterology, University of Palermo), Carolina Ciacci (Dept. Medicine and Surgery, University of Salerno), Cinzia D’Agate (Gastroenterology and Digestive Endoscopy, University of Catania), Italo De Vitis (Internal Medicine and Gastroenterology, Catholic University, Rome), Antonio Di Sabatino (First Dept Internal Medicine, University of Pavia), Gianmarco Fava, (Gastroenterology and Digestive Endoscopy, University of Ancona), Maria Rita Frau (Pediatrics, S. Francesco Hospital, Nuoro), Alessandro Fugazza (Internal Medicine and Gastroenterology, Civil Hospital, Codogno), Stefano Andrea Grassi (Gastroenterology, Civil Hospital, Bassano del Grappa), Giuseppina Larcinese (Civil Hospital Lanciano, Italy), Giovanni Latella (Gastroenterology, University of L’Aquila), Adriano Lauri (Gastroenterology and Digestive Endoscopy, Civil Hospital, Pescara), Angelo Lauria (Gastroenterology and Digestive Endoscopy, Bianchi-Melacrino-Morelli Hospital, Reggio Calabria), Nicoletta Lenoci (Gastroenterology, Valduce Hospital, Como), Norma Beatriz Lopez Rios (Pediatrics, Civil Hospital, Sondrio), Donatella Macchia (Allergology and Clinical Immunology, S. Giovanni di Dio Hospital, Florence), Mauro Minelli (Allergology and Clinical Immunology, Anthea Hospital, Bari), Beba Molinari (Observatory of the Italian Coeliac Association, Genoa), Olivia Morelli (Gastroenterology and Hepatology, University of Perugia), Maria Gloria Mumolo (Gastroenterology, University of Pisa), Giovanni Niccoli (Gastroenterology, Civil Hospital, Livorno), Caterina Pacenza (Pediatrics, S.Giovanni di Dio Hospital, Crotone), Francesco Pallone (Gastroenterology, Torvergata University, Rome), Fabrizio Parente (Gastroenterology, Alessandro Manzoni Hospital, Lecco), Raffaella Pulitanò (Gastroenterology and Digestive Endoscopy, Civil Hospital, Cuneo), Rossella Pumpo (Digestive Endoscopy, Loreto Mare Hospital, Naples), Gabriele Riegler (Gastroenterology and Digestive Endoscopy, University of Naples), Antonio Rispo (Gastroenterology and Hepatology, University of Naples), Flavio Romolo Rispoli (Civil Hospital, Sulmona, Italy), Francesco Tedone (Gastroenterology, Civil Hospital, Grosseto), Flavio Valiante (Gastroenterology, Civil Hospital, Feltre), Giovanni Viviani (Gastroenterology and Digestive Endoscopy, Civil Hospital, Manerbio), Paolo Usai Satta (Gastroenterology, Brotzu Hospital, Cagliari).

## Pre-publication history

The pre-publication history for this paper can be accessed here:

http://www.biomedcentral.com/1741-7015/12/85/prepub

## Supplementary Material

Additional file 1 Questionnaire for non-celiac gluten sensitivity (NCGS) prospective survey promoted by the Italian Association for Celiac Disease (AIC) and Celiac Foundation (FC). Click here for file
