# Supplementary material for: An Italian prospective multicenter survey on patients suspected of having non-celiac gluten sensitivity
Source: BMC Med. 2014 May 23;12:85. doi: 10.1186/1741-7015-12-85 (PMC4053283; doi:10.1186/1741-7015-12-85)
Supplement: Additional file 1 — Questionnaire for non-celiac gluten sensitivity (NCGS) prospective survey promoted by the Italian Association for Celiac Disease (AIC) and Celiac Foundation (FC). [file 1741-7015-12-85-S1.docx]

**Questionnaire for Non-Celiac Gluten Sensitivity (NCGS) prospective survey promoted by the Italian Association for Celiac Disease (A.I.C.) and Celiac Foundation (F.C.)**

1. Center Affiliation, including Specialization:

2. Region (Italy):

3. Name of the investigator in charge of the Center:

4. Patient’s age (years):

5. Gender

Female

Male

**6-33 Symptoms/Signs (Related to gluten ingestion)**

6. Tiredness

Yes

No

7. Lack of well being

Yes

No

8. Weight loss

Yes

No

9. How much?

Kg. (number)

10. Recurrent aphthous stomatitis

Yes

No

11. Heartburn

Yes

No

12. Acid regurgitation

Yes

No

13. Gastric pain

Yes

No

14. Nausea

Yes

No

15. Aerophagia

Yes

No

16. Bloating

Yes

No

17. Abdominal pain

Yes

No

18. Diarrhea

Yes

No

Number of evacuations per day:

19. Constipation

Yes

No

20. Alternating bowel habits

Yes

No

21. Anaemia

Yes

No

22. Headache

Yes

No

23. Numbness

Yes

No

24. Foggy mind

Yes

No

25. Myalgia

Yes

No

26. Arthralgia

Yes

No

27. Depression

Yes

No

28. Anxiety

Yes

No

29. Asthma

Yes

No

30. Rhinitis

Yes

No

31. Skin rash

Yes

No

32. Dermatitis

Yes

No

33. Any other symptom?

Specify:

34. Frequency of symptoms in relationship with gluten ingestion?

Always

Often

Occasionally

35. Time interval after gluten ingestion

Within 6 hours

Between 6 and 24 hours

After 24 hours

36. How long were the symptoms present before NCGS detection?

1 month

6 months

> 6 months

**37-42 Associated Disorders**

37. Eating behavior disorder

Yes

No

Which?

38. Irritable bowel syndrome (IBS) according to Rome III criteria

Yes

No

39. Food intolerance

Yes

No

Which?

40. Allergy

Yes

No

Which?

41. Psychiatric disorder

Yes

No

Which?

42. Autoimmune disorder

Yes

No

Which?

43. Family history of Celiac Disease

Yes

No

**44-49 Who was the first to suspect NCGS?**

44. Patient (self-diagnosis)

Yes

No

45. Friends

Yes

No

46. Chemist

Yes

No

47. General practitioner

Yes

No

48. Gastroenterologist

Yes

No

49. Omeopath

Yes

No

**50-59 Laboratory Data**

50. Antigliadin antibody of IgG class

Positive

Negative

Not done

51. Antigliadin antibody of IgA class

Positive

Negative

Not done

52. Deamidated gliadin peptide antibody of IgG class

Positive

Negative

Not done

53. Deamidated gliadin peptide antibody of IgA class

Positive

Negative

Not done

54. Total serum IgE

Normal

Increased

Not done

55. RAST/PRICK to gluten/wheat

Positive

Negative

Not done

56. RAST/PRICK to other allergens

Positive

Which allergen/s?

57. Biochemical abnormalities

Present

Absent

Which?

58. HLA-DQ2

Present

Absent

Not done

59. HLA-DQ8

Present

Absent

Not done

60. Duodenal biopsy

Performed

Not performed

Lesion type (Marsh-Oberhüber classification):
